# Interview with the World Class Authorities Frontiers of Cancer Research: An exclusive interview with Professor Luis Diaz

**DOI:** 10.1002/ags3.70003

**Published:** 2025-03-03

**Authors:** Haruna Takeda, Koshi Mimori, Masanobu Oshima, Ken Shirabe, Yuko Kitagawa

**Affiliations:** ^1^ The Laboratory of Molecular Genetics National Cancer Center Research Institute Tokyo Japan; ^2^ Department of Surgery Kyushu University Beppu Hospital Beppu Japan; ^3^ WPI Nano‐Life Science Institute (Nano‐LSI) Kanazawa University Kanazawa Japan; ^4^ Division of Genetics, Cancer Research Institute Kanazawa University Kanazawa Japan; ^5^ Department of General Surgical Science, Graduate School of Medicine Gunma University Maebashi Japan; ^6^ Department of Surgery Keio University School of Medicine Tokyo Japan

**Keywords:** circulating tumor DNA (ctDNA), colorectal cancer (CRC), immune checkpoint inhibitors (ICI), liquid biopsy technologies, microsatellite instability (MSI‐H), mismatch repair deficiency (dMMR), PD‐1 blockade, precision oncology

## Abstract

This manuscript provides an in‐depth interview with Prof. Luis Diaz, Head of Oncology at Memorial Sloan Kettering Cancer Center and Editor‐in‐Chief of *Cancer Discovery*. A globally recognized leader in oncology, Prof. Diaz discusses the transformative impact of precision oncology, particularly the role of mismatch repair deficiency (dMMR) and microsatellite instability‐high (MSI‐H) biomarkers in immunotherapy. He highlights the groundbreaking success of PD‐1 blockade therapies, such as dostarlimab, which have achieved unprecedented complete response rates in dMMR/MSI‐H rectal cancer, emphasizing its tumor‐agnostic potential. Prof. Diaz reflects on the evolution of cancer diagnostics, notably circulating tumor DNA (ctDNA) for minimal residual disease (MRD) detection, and its implications for treatment personalization and early detection. He also addresses the challenges and prospects of cancer prevention, advocating for innovative approaches such as immunoprevention and vaccines targeting tumor‐specific pathways, like the HPV vaccine for cervical cancer. The interview underscores the importance of fundamental research in advancing cancer care and the necessity of interdisciplinary collaboration to address unresolved questions in tumor biology. By sharing his vision and pioneering achievements, Prof. Diaz inspires the next generation of clinicians and researchers to pursue bold innovations, ultimately aiming to enhance patient outcomes and revolutionize the future of oncology. This dialogue serves as a significant resource for understanding current trends and future directions in cancer research and treatment.

## Graphical abstract

This is the second installment in the series, “The Interview with the World‐Class Authorities.” We are honored to welcome Prof. Luis Diaz, the Director of the Division of Solid Tumor Oncology at Memorial Sloan Kettering Cancer Center and Editor‐in‐Chief of *Cancer Discovery*. Prof. Diaz's illustrious career has been distinguished by groundbreaking contributions to cancer genomics, immunotherapy, and liquid biopsy technologies. His landmark research on PD‐1 blockade in mismatch repair‐deficient cancers has revolutionized the treatment paradigm for gastrointestinal and other solid tumors. Among his many accolades are the AACR Waun Ki Hong Award and recognition as a Clarivate Highly Cited Researcher. Prof. Diaz's remarkable achievements and unwavering idealism continue to inspire not only young surgical scientists but also aspiring clinical surgeons.

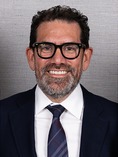

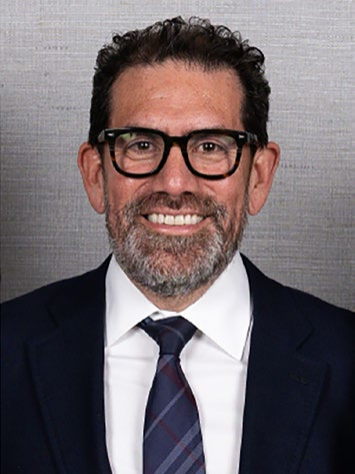

**Luis Alberto Diaz, Jr., M.D**.

Dr. Luis Diaz is an internationally celebrated physician‐scientist renowned for his groundbreaking contributions to oncology. He worked under the mentorship of Dr. Bert Vogelstein, a leading figure in cancer genetics (2002–2004). Currently, Dr. Diaz heads the Division of Solid Tumor Oncology at Memorial Sloan Kettering Cancer Center, holding the prestigious Grayer Family Chair in Medicine (2016–present), and serves as a Professor of Medicine at Weill Cornell Medical College (2019–present).

Dr. Diaz's career has been marked by pioneering achievements in cancer genomics, immunotherapy, and liquid biopsy technologies. Notably, his seminal research on PD‐1 blockade in mismatch repair‐deficient cancers has reshaped the treatment landscape for gastrointestinal and other solid tumors, earning him accolades such as the AACR Waun Ki Hong Award (2020) and Clarivate Highly Cited Researcher recognition (2018).

A visionary in precision oncology, Dr. Diaz co‐founded Personal Genome Diagnostics, now part of LabCorp, advancing personalized cancer diagnostics globally (2011–2022). His leadership extends to chairing prestigious advisory boards, including the National Cancer Advisory Board under the former president Joe Biden (2021‐), and serving as Editor‐in‐Chief of *Cancer Discovery* (2018–present).

Honored by the National Academy of Medicine and the AACR Academy (2023), Dr. Diaz is a luminary in cancer care, dedicated to improving patient outcomes through innovation, mentorship, and collaboration. His work continues to inspire the next generation of oncology breakthroughs.

## 1 OPENING REMARK

This program is proudly sponsored by the Japanese Society of Gastrointestinal Surgery and the Japanese Cancer Association. It features a special interview with leading experts and distinguished researchers from around the world. Today, we are honored to welcome Dr. Luis Diaz from Memorial Sloan Kettering Cancer Center. Joining as interviewers are Koshi Mimori from Kyushu University, Beppu Hospital and myself. We will begin by asking Prof. Diaz several questions. It is a pleasure to have you here. Welcome to the program and thank you for joining us.

### 1.1 Question #1: The effect of PD‐1 blockade on dMMR‐MSI‐H CRC

#### 1.1.1 What are the clinical characteristics of tumors and patients with mismatch repair deficient and MSI high tumors that do not respond to PD‐1 blockade?

**H.T.:** Okay, I would like to ask you about the effect of PD‐1 blockade on tumors with high MSI. PD‐1 blockade has shown remarkable effects in mismatch repair‐deficient and MSI‐high CRC patients, as many of them show a complete response. What genetic mutations are likely to serve as antigens in mismatch repair‐deficient and MSI‐high CRC? Is there a risk of recurrence 5 or 10 years after treatment? What are the clinical characteristics of tumors and patients with mismatch repair‐deficient and MSI‐high tumors that do not respond to PD‐1 blockade?

**Prof. Diaz:** These are excellent questions. And when we began to look at this question many years ago, it was simply with a hypothesis that tumors with many mutations would respond better than tumors with low mutations. And we knew that tumors with mismatch repair deficiency, an inactivation of mismatch repair in one of four different genes would result in a tumor that had a very high mutational burden. And we hypothesize that if those mutations were expressed and transcribed, that they would look foreign to the immune system and if we give anti‐PD‐1, there may be a powerful anti‐tumor response against the tumor.

We conducted the study on patients with metastatic colorectal cancer and what we found was the majority of patients not only had the shrinkage of tumor but in half the patients they were cured over time, and these were patients with metastatic disease. We then went on to say if we didn't look at patients with metastatic disease who are very advanced and we looked at them when they are first diagnosed with metastatic disease, could they do well, and we found that they did. But then there was another question, could these patients with mismatch repair deficiency and high mutation burden if they warrant colon cancer,^1^ let's say, their pancreas,^2^ or ovary,^3^ or endometrial,^4^ or cholangio,^5^ or bladder,^6^ would they respond? And what we found was that the answer was yes, and so it was a tumor agnostic biomarker.

It did not matter where the tumor was born, it mattered more the genetics of the tumor. More recently what we looked at was could we take the same approach and look prior to treatment, so in the early‐stage disease. And we are very surprised to find that unlike in the metastatic disease where we can cure half the patients, we could actually cure 100% of the patients with a simple treatment with anti‐PD‐1.^7^, ^8^ And so I think that clinically it shows that this is a very powerful biomarker, and it shows a very strong response to PD‐1 blockade.

But the question is, are there any shared targets or biomarkers across these? And we are not finding many. It is more the class of the type of new antigen, which are likely insertion deletions, but a specific type of insertion deletions frame shifts, those insertion deletions that are not divisible by three. And so, we think that's the primary target and why we are seeing such a strong response in these tumors.

#### 1.1.2 Can driver genes like APC, KRAS, p53 be a good neoantigen?

**K.M.:** Not every gene can be a neoantigen, correct? I would like to confirm whether driver genes like APC, KRAS, and p53 are unlikely to serve as good neoantigens, is that right?

**Prof. Diaz:** Yeah. Well, I think that is a very good question. We know for many years of cancer research, there are two types of mutations that occur. One that are activating mutations and one that are suppressing mutations. When you have an activated mutation—and there is a third one, but I will talk about that in second—it is expressed. And oftentimes it can be amplified, or the mutation is gain of function.

And oftentimes it happens in kinases or signaling pathways. The other type of mutation turns genes off. And when you turn a gene off, it is not expressed. If it is not expressed, it cannot be a neoantigen. So, it has to be expressed. And then it has to bind to the HLA very tightly. But let's talk about the third mutation. What about irrelevant mutations, passenger mutations? One that may not affect the function, are still expressed, and look foreign. Those can also be neoantigens. I think that those are that very important distinction. Now, certainly, APC or p53 could be neoantigens if they are expressed. And so I think that's a major difference.

#### 1.1.3 Can the chemotherapy or radio therapy elevate immunogenicity (abscopal effect)?

**K.M.:** Clinically, we are conducting chemotherapy or radiotherapy. These treatments are toxic to cancer cells, and we expect that they may produce neoantigens. What are your thoughts on this? Chemotherapy acts on cancer cells, potentially producing neoantigens, which could enhance the immune response and lead to further cancer cell destruction.

**Prof. Diaz:** Yeah. The goal of chemotherapy is to kill the cancer. But if it doesn't, and it actually causes mutations that are immunogenic, then potentially that can cause a tumor that is more immunogenic. I think one of the problems is, or one of the questions is, most mutations are not immunogenic. And at this meeting, which you have organized, we have seen some fantastic talks today about mutation of signatures. And there was another talk on inducing mutational signatures, looking at mutational signatures in animals. The drugs cause many different types of mutational signatures and different types of mutations, and most are not immunogenic.

#### 1.1.4 Are there any specific sequences then needed to be presented on the cellular surface?

**H.T.:** The specific sequences then needed to be presented to the surface.

**Prof. Diaz:** Yeah, and so they have to look the most foreign. And so, there is something that we looked at called a blast algorithm that tells you if two proteins are related, right, by the sequence homology, we took that and looked at it reverse. The more different a peptide sequence is, the more likely it is going to be foreign. And that has to be the case there. I am sure you use blasts all the time, right? Yeah.

#### 1.1.5 Does the dostarlimab, PD‐1 inhibitors effective rectal cancer specifically?

**K.M:** 1.1.5 mentioned that dostarlimab, a PD‐1 inhibitor, is highly effective in rectal cancer, achieving a 100% complete response. That is truly amazing and sensational on a global scale. We are reading about it with great enthusiasm. Is this remarkable effect observed specifically in rectal cancer, or can similar outcomes be seen in other parts of the colon as well? Or is this phenomenon unique to the rectum? Could you elaborate on this?

**Prof. Diaz:** That is a great question. And you are exactly right. Dostarlimab, an anti‐PD‐1 agent, in rectal cancer that was mismatch repair deficient, we saw 100% response.^8^ Obviously, it has never been done before in cancer ever, right? We were very excited. We now have treated almost 100 patients the same way, and we are seeing the same thing. And what's important is the patients don't need chemotherapy, they don't need radiation and no surgery. It's, I think, a remarkable finding.

The question that you have is outstanding. What about in the colon or in the biliary tract or in the pancreas or in the esophagus or epigastric or the prostate or bladder?

And so, we have treated patients like this. And what we are finding is that we have patients who have a complete response as well and don't need surgery. We haven't published that yet, but I am telling you unpublished information.

#### 1.1.6 What is the secret of Dostarlimab implemented 100%CR for rectal cancer?

**K.M.:** What is the secret behind the drug's success? Is dostarlimab one of the key factors behind this breakthrough?

**Prof. Diaz:** No. Is the drug important? We don't know. Certainly, the drug is very good. But it could be something that all the anti‐PD‐1s show, or it could be just that one drug. But it tells us it's very exciting because it changes lives of the patients. Because if you can imagine, as a surgeon you know, many surgeries change the life of a patient, whether it's removal of a major organ or just the risk of surgery. It's life altering. And the patient feels it. And if we can change that, then we think the patient will be much happier.

**K.M**.: Is this a game changer, right?

**Prof. Diaz:** Game changer, yeah.

### 1.2 Question #2: Most exciting findings in your research career

#### 1.2.1 What is your most exciting research topic at present?

**KM:** What has been the most exciting result of your research so far?

**Prof. Diaz:** That's a fantastic question. And I have been lucky because I have had many years of research that I have been involved with. I think if I think back to work with my mentor, Bert Vogelstein, one of the most exciting things was showing that circulating mutations in the blood not only could be detected, but they would go up as a tumor grew, and they would go down as a tumor responded.^9^ And they would disappear when you cut the tumor out.

And we first showed that in mice and then we showed that in humans. We showed that we could track resistance. We showed that we could detect minimal residual disease. And then eventually we showed that you can use it for early detection. And I think that was one exciting chapter. Another exciting chapter was when we looked at giving live bacteria to patients to destroy the tumor. And that was a very exciting era as well. And then more recently looking at the determinants of immunogenicity, like, mismatch repair deficient tumors ensuring that immunotherapy could work quite potently there.

But what I think that the future is in—what I think is probably the most exciting data because it can help the most, is the data on using the immune system to prevent cancer. And this is work that was led by Zsofia Stadler, but we were working on this closely, where we looked at patients with Lynch syndrome who developed mismatch repair deficient colon cancer.^10^ What if they gave them anti‐PD‐1? And what we showed was that you could delay or prevent cancers in those patients. I think that, for me, that piece of data is very exciting because it opens the door to prevention of cancer. And if we look at what we understand about the development of cancer, both in mice models and in human models, there may be a point where our therapies work better earlier than in the advanced stages. And I think, to me, that's the most exciting data I have seen in a long time.

#### 1.2.2 Despite the preventive usage of PD‐L1 blockade, skin cancer is growing and vascular organ tumors are down, how do you elaborate on this finding

**K.M.:** I am curious to know that Zsofia Stadler showed that after the period of inhibition, later, skin cancer is growing and vascular organ tumors are down, right? How do you elaborate on this finding?

**Prof. Diaz:** I think it has something, again, to do with the fact the immune system behaves very differently in different parts of the body. For instance, inside visceral organs, the immune system is more inhibited whereas in barriers like the skin or the mucosa, the immune system may be more potent to make sure that things don't get in. When you inhibit the immune system, you are able to eradicate or prevent or delay the growth of tumors in visceral tissues. But there may be an immune suppression that allows the development of non‐dangerous cutaneous tumors.

It's something that we have to look at, and I think that highlights the difference between the immune system within our bodies and the immune system in barrier structures.

#### 1.2.3 Why do germline mutations, which affect the whole body, manifest specifically in the skin?

**K.M.:** Germline has mutated. It is probably, I guess, the skin cell has mutated, right? It's related to the result. Maybe, there is a specific event in skin. Why does it happen only specifically in the skin?

**Prof. Diaz:** Yeah, it's a fantastic question. And we do not know the answer, right? But it may be that, paradoxically, rather than anti‐PD‐1 being protecting for tumor growth, in that setting, it may have the opposite effect.

Anyway, PD‐1 inhibition administration could extend recurrence of RFS period, the period of the other cancers. It may suppress the cancers that are lethal, but it may put you at higher risk for cutaneous cancers.

#### 1.2.4 Can the circulating DNA apply to the treatment?

**K.M.:** The first one is circulating tumor DNA.^11^ It's just a tool for diagnosis. But you mentioned that it might be a therapeutic agent or approach that utilizes circulating DNA in treatment?

**Prof. Diaz:** I think what's exciting here is that the use of circulating tumor DNA to determine whether or not there is residual tumor after surgery is a big possibility. And in the event that there is circulating tumor DNA detected after surgery, what we found in many studies now is that means that there is tumor remaining in the body and that the tumor will come back.

And if the circulating tumor DNA is negative, it's likely that the tumor will not come back. So, how can that influence therapy? Well, if it's positive, it means that we have to treat, because it's going to come back. Because the half‐life (of ctDNA) is 2 h. So, by 10 h after you cut it out, it should be gone. It is not like a protein biomarker, which has a 2‐week half‐life.

And so that means it would take probably for it to go away in the order of 10 weeks, right, completely, maybe faster, but with circulating tumor DNA, the half‐life is an advantage because it allows us to see what's going on immediately. If it's positive, you should act. But what if it's negative? Maybe, you can stop chemotherapy.

#### 1.2.5 How do you think about the roles of adjuvant chemotherapy?

**Prof. Diaz:** If you think about colon or breast or lung cancer, where everyone is getting chemotherapy after the tumor is resected, about 80% of those patients don't need the chemotherapy.

And so, I think that this will allow us to remove that at some point. And at this meeting, there was some very interesting data and very interesting study designs that I think will be very, very useful for sure. These are excellent and encouraging remarks for patients experiencing adverse effects.

### 1.3 Question #3: What's your message to the younger researchers?

#### 1.3.1 Message to young basic researchers

**H.T.:** I am more focused on basic medical research, and I truly respect you because you excel in both roles—as a clinician and a basic medical researcher. What message would you like to share with younger researchers? Should they fully focus on basic medical research, or should they aim to balance both clinical practice and research?

**Prof. Diaz:** I think it's a very good question. I think we need more basic research, much more basic research.

**H.T.:** Your words really encourage us.

**Prof. Diaz:** And I think we need to discover new pathways, new mechanisms of action, new biological insights. The translation probably happens too much. I think we need to do more fundamental work and less translation at this point because most things are not ready to be translated. That said, you never know. So, maybe, you do have to take chances sometimes as well. But I would say to people in the laboratory, focus on answering a question that has never been answered before. Don't try to just repeat the work of someone else. Focus on answering questions that have never been answered before. Take a risk.

**K.M.:** Your words really encourage us. Without taking risk, we cannot get anything, right?

**Prof. Diaz:** Correct.

### 1.4 Question #4: Prevention of cancers

#### 1.4.1 How do we prevent cancers?

**Prof. Diaz:** I think that's a very important question, but it's also a very hard question. If we think about the causes of cancer, there are only a few actually, one of them are hereditary mutations that are passed on in families. Another one is environmental exposures—tobacco or UV light or toxic chemicals. Another one is we believe inflammation or increasing the doubling time of tumors. Another one is age because as we get older, the cells have more mutations and the higher chance of having mutation. And the final one is the toughest one, bad luck. Spontaneous mutation in the wrong gene, in the wrong location, in the wrong organ. If you look at those causes, how can we address them? Well, the ones that are caused by environment or external toxins like radiation, or UV light, or tobacco, you avoid that. And we are already seeing benefits from that. We are seeing lung cancer rates going down because of smoking cessation. We knew people putting sunscreen on, decreases the risk of skin cancer. So, that one is easy. If patients have a family history of a manageable syndrome, we screen them.

For early detection and inflammation, we try to lower the inflammation. Now, we don't know if that is beneficial or not, but that's one possibility. But what about bad luck and age? We don't have a good strategy. One strategy is, can we develop tests for early detection of cancer? And I think that's very exciting, but we are working on that. But an even more interesting one would be is, can we detect the risk factor and then modify the development of that cancer, so prevent the development of that cancer.

And so, I think that's going to be the next big thing. Yes, we can look at cancers, we can understand their genetic mutations, we can treat, but we are still not curing many patients.

We can detect it earlier and cut it out, but that has a lot of morbidity. Can we prevent the cancer from happening? And I think strategies around chemoprevention, immunoprevention will be a big one. But one of the aspects of immunoprevention, which I think is going to be very interesting, is treatment with vaccines, either treating—oh, and I forgot one—infectious agents, treating them with the vaccine to treat the etiology of that tumor. And so, probably, the most successful thing in all of cancer is the HPV vaccine. HPV causes cervical cancer, vaginal cancer, penile cancer, vulvar cancer, anal cancer, and tonsil cancer. Is there a way that we could give a vaccine against HPV and prevent the cancer? And the answer is yes.

It's incredible, right? I think something similar has to happen across tumor types. It's not easy. And it's going to take a lot of fundamental basic science and understanding what pathways we attack with the vaccine in order to have the biggest impact.

#### 1.4.2 Can mutation signatures predict cancers?

**KM:** You mentioned that bad luck and aging can cause mutations. We would like to understand the risks associated with the signatures of these mutations in normal, healthy samples. Is it possible to assess or analyze this?

**Prof. Diaz:** Yeah, I think mutational signatures will play a big role. But even before that, I think behavioral risk. If someone is a smoker, can we prevent with vaccine? Or if somebody goes out in the sun a lot, can we prevent that risk? And we will identify other risk factors, whether it's by mutational signatures or other things, that I think we can tailor the vaccines for them.

**KM:** It's very difficult to know that in human beings, the signatures are very difficult because we have many numbers of signatures come from the animal model, so very high exposure of carcinogen (it would require a significantly higher level of exposure to human compared to animal models). So, it is easy to identify signatures not in human but in animal models.

**Prof. Diaz:** Yeah. I don't think it's going to all be mutational signatures. But I do think there will be some that will be important to us.

#### 1.4.3 Is there still a window for MSS tumors using ICI combined with other drugs?

**HT:** More than 80% of tumors carry mutations in APC. I understand that immune checkpoint inhibitors (ICI) are effective in MSI‐high tumors. However, the majority of colon cancers are MSS, which are driven by APC inactivation. I am wondering if there is still a therapeutic window for treating MSS tumors using ICI in combination with other drugs?

**Prof. Diaz:** I think tumors at our early stage will see benefit there. Whether the Wnt pathway, either β‐catenin signaling, or the inactivation of APC or even RNF43 is involved, I think, all of those will be factors in determining whether immunotherapy works or not. I think we need more understanding of the pathway. It's a very important pathway.

**HT:** This is very important. Some studies using animal models have shown that combining PD‐1 blockade with TGFβ inhibitor blockade is effective in MSS mouse models.^12^ Similarly, other drug combinations have also demonstrated efficacy in mouse models.^13^

**Prof. Diaz:** I think we have the anti‐PD‐1, now we need a good anti‐TGFβ agent to show that. And whether you target the receptor or the circulating molecule is unknown.

#### 1.4.4 Do you think that TGFβ blockade or TGFβ activation will impact on the cancer treatment?

**HT:** I would like to ask if there is any clinical evidence regarding the use of TGFβ blockade in treating TGFβ‐activated tumors or similar conditions.

**Prof. Diaz:** Yeah, we don't have the good data yet, but I think that's a very important question to answer in the clinic.

#### 1.4.5 How do you think about the cost for cancer prediction or prevention among bunch of cohorts?

**KM:** Okay, so we are discussing cancer prevention. We understand that blood tests can detect cancers by identifying mutations in cell DNA, which is often found in cancer patients. However, to prevent cancers, we need to screen large cohorts, including many healthy individuals. This brings up the issue of cost for screening these cohorts. Currently, sequencing of cell free DNA testing remains expensive, costing as much as ¥100 000 in Japan. So, what measures can we use to detect cancer in healthy individuals more effectively and affordably?

**Prof. Diaz:** I think that's a very good question because I don't think the tests are good enough yet. So, we have to study those tests in healthy donors to see if that has effect.

#### 1.4.6 What is the best tool for MRD?

**Prof. Diaz:** Cell‐free DNA. Whether it's mutations or methylation or any other changes or fragmentomics. But what I would say is that we are going to have to look at the outcome of survival. Does the biomarker impact survival? And I think that's going to be a big outcome to look at. We can't just look at just detecting cancer.

**KM:** So, you think that mutated cell free DNA should be the best marker to MRD?

**Prof. Diaz:** For MRD, probably yes. It's going to be more sensitive especially when it's informed by the tumor. For early detection, we don't know yet, whether it will be methylation or mutations or fragmentomics or something else. I think it's going to be a combination. I think there will have to be a subsequent clinical CT scan to really understand that data well.

#### 1.4.7 In terms of the sequential order of driver genes in the classical pathway, such as APC, KRAS, and TP53, are there any biological mechanism to explain it?

**KM:** And a related question about bad luck or aging. APC mutations were followed by KRAS mutations and TP53 mutations in colon cancer. Why do these mutations occur sequentially? Does APC mutation cause KRAS mutation, or do both mutations lead to TP53 mutation? How is this sequential progression determined?

**Prof. Diaz:** So, it looks like a line, but it's really bad. It's really stochastic. Otherwise, we would have many tumors.

**KM:** For example, in colon cancer, does the sequence of APC mutation followed by KRAS mutation and then TP53 mutation occur purely by probability, or is there a biological mechanism behind it?

**Prof. Diaz:** No, it probably is. But they occur randomly. But it appears like the sequential.

### 1.5 Question #5: Association of the inflammation and colon cancer

#### 1.5.1 Do you believe that the colon cancer with inflammation showed clinical benefit for the ICI?

**HT:** I am also focusing on the association between inflammation and colon cancer. Inflammation involves various inflammatory cytokines, such as IFNγ and TNFα, which lead to high expression of PD‐1 or PD‐L1. Do you believe that colon cancer associated with inflammation shows a clinical benefit from immune checkpoint inhibitors (ICIs)?

**Prof. Diaz:** Yeah, so we have not seen a lot of benefit with inflammatory‐related cancers. It's interesting. But we do know that inflammation causes a higher proliferation rate, a higher cellular turnover, and therefore a higher incidence of cancer. Whether or not that will be led to a state that's more PD‐1 sensitive, we have not seen that yet. But I think it's a very good question.

## 2 CLOSING REMARKS

We extend our deepest gratitude to Prof. Luis Diaz for sharing his invaluable insights and groundbreaking research with us today. His expertise has illuminated critical aspects of immunotherapy, cancer prevention, and the underlying mechanisms of tumor biology, offering profound inspiration and guidance to the global medical and research community. Prof. Diaz's pioneering work exemplifies the transformative potential of innovative science and its application to improving patient outcomes.

This interview has not only enriched our understanding of cancer research but also underscored the importance of fostering an interdisciplinary approach to tackling this complex disease. As Prof. Diaz aptly highlighted, the journey to groundbreaking discoveries is paved with curiosity, perseverance, and the courage to take risks. We hope this exchange inspires young researchers and clinicians worldwide to continue advancing the frontiers of science and medicine. On behalf of the Japanese Cancer Association and the Japanese Society of Gastrointestinal Surgery, we extend our heartfelt thanks to all who contributed to making this program a resounding success.

## AUTHOR CONTRIBUTIONS

**Haruna Takeda:** Writing – original draft. **Masanobu Oshima:** Supervision. **Koshi Mimori**: an interviewer. **Ken Shirabe:** Supervision. **Yuko Kitagawa:** Conceptualization and Supervision.

## CONFLICT OF INTEREST STATEMENT

The authors declare no conflicts of interest for this article.

## ETHICS STATEMENT

Approval of the Research Protocol by an Institutional Reviewer Board: N/A.

Informed Consent: N/A.

Registry and the Registration No. of the study/trial: N/A.

Animal Studies: N/A.

## References

[1] Chalabi M , Verschoor YL , Tan PB , Balduzzi S , Van Lent AU , Grootscholten C , et al. Neoadjuvant immunotherapy in locally advanced mismatch repair‐deficient colon cancer. N Engl J Med. 2024;390(21):1949–1958. 38838311 10.1056/NEJMoa2400634

[2] Bian J , Almhanna K . Pancreatic cancer and immune checkpoint inhibitors‐still a long way to go. Transl Gastroenterol Hepatol. 2021;6:6. 33409400 10.21037/tgh.2020.04.03 PMC7724172

[3] Nonomura Y , Nakayama K , Nakamura K , Razia S , Yamashita H , Ishibashi T , et al. Ovarian Endometrioid and clear cell carcinomas with low prevalence of microsatellite instability: a unique subset of ovarian carcinomas could benefit from combination therapy with immune checkpoint inhibitors and other anticancer agents. Healthcare (Basel). 2022;10(4):694. 35455871 10.3390/healthcare10040694 PMC9032309

[4] Khushman MM , Toboni MD , Xiu J , Manne U , Farrell A , Lou E , et al. Differential responses to immune checkpoint inhibitors are governed by diverse mismatch repair gene alterations. Clin Cancer Res. 2024;30(9):1906–1915. 38350001 10.1158/1078-0432.CCR-23-3004

[5] Yang X , Lian B , Zhang N , Long J , Li Y , Xue J , et al. Genomic characterization and immunotherapy for microsatellite instability‐high in cholangiocarcinoma. BMC Med. 2024;22(1):42. 38281914 10.1186/s12916-024-03257-7 PMC10823746

[6] Chandran EBA , Iannantuono GM , Atiq SO , Akbulut D , Sinaii N , Simon NI , et al. Mismatch repair deficiency and microsatellite instability in urothelial carcinoma: a systematic review and meta‐analysis. BMJ Oncol. 2024;3(1):e000335. 10.1136/bmjonc-2024-000335 PMC11203074 39086924

[7] Cercek A , Diaz LA Jr . PD‐1 blockade in mismatch repair‐deficient rectal cancer. Reply. N Engl J Med. 2022;387(9):855–856. 10.1056/NEJMc2209706 36053518

[8] Cercek A , Lumish M , Sinopoli J , Weiss J , Shia J , Lamendola‐Essel M , et al. PD‐1 blockade in mismatch repair‐deficient, locally advanced rectal cancer. N Engl J Med. 2022;386(25):2363–2376. 35660797 10.1056/NEJMoa2201445 PMC9492301

[9] Diaz LA Jr , Williams RT , Wu J , Kinde I , Hecht JR , Berlin J , et al. The molecular evolution of acquired resistance to targeted EGFR blockade in colorectal cancers. Nature. 2012;486(7404):537–540. 22722843 10.1038/nature11219 PMC3436069

[10] Harrold EC , Foote MB , Rousseau B , Walch H , Kemel Y , Richards AL , et al. Neoplasia risk in patients with lynch syndrome treated with immune checkpoint blockade. Nat Med. 2023;29(10):2458–2463. 37845474 10.1038/s41591-023-02544-9 PMC10870255

[11] Kotani D , Oki E , Nakamura Y , Yukami H , Mishima S , Bando H , et al. Molecular residual disease and efficacy of adjuvant chemotherapy in patients with colorectal cancer. Nat Med. 2023;29(1):127–134. 36646802 10.1038/s41591-022-02115-4 PMC9873552

[12] Tauriello DVF , Palomo‐Ponce S , Stork D , Berenguer‐Llergo A , Badia‐Ramentol J , Iglesias M , et al. TGFbeta drives immune evasion in genetically reconstituted colon cancer metastasis. Nature. 2018;554(7693):538–543. 29443964 10.1038/nature25492

[13] Yang B , Li X , Fu Y , Guo E , Ye Y , Li F , et al. MEK inhibition remodels the immune landscape of mutant KRAS tumors to overcome resistance to PARP and immune checkpoint inhibitors. Cancer Res. 2021;81(10):2714–2729. 33589518 10.1158/0008-5472.CAN-20-2370 PMC8265237

